# Prognostic factors for lymph node metastasis from advanced squamous cell carcinoma of the skin of the trunk and extremities

**DOI:** 10.1186/1477-7819-6-73

**Published:** 2008-07-04

**Authors:** Vinicius de Lima Vazquez, Teoclito Sachetto, Natalia Martins Perpetuo, Andre Lopes Carvalho

**Affiliations:** 1 Department of Surgery, Hospital de Cancer de Barretos, Rua Antenor Duarte Villela, 1331 Barretos – SP, 14784-400, Brazil; 2 Department of pathology, Hospital de Cancer de Barretos, Rua Antenor Duarte Villela, 1331 Barretos – SP, 14784-400, Brazil

## Abstract

**Background:**

Squamous cell carcinoma (SCC) of the skin of the trunk and extremities may present lymph node metastasis with difficult disease control and poor survival. The purpose of this study was to identify risk factors for lymph node metastasis and outcome.

**Patients/Methods:**

Retrospective review of 57 patients with locally advanced SCC of the trunk and extremities was performed and several clinical variables including age, gender, ethnicity, previously injured skin (burns, scars, ulcers and others), patient origin (rural or urban), anatomic site and treatment were studied.

**Results:**

Fifteen patients presented with previous skin lesions. Thirty-six were classified as T3 tumors and 21 as T4; 46 were N0, and 11, N1. Eleven N0 patients presented lymph node metastasis during follow up. Univariate analysis identified previous skin lesions (ulcers and scars) as risk factor for lymph node metastasis (p = 0.047). Better survival was demonstrated for T3 (p = 0.018) classification. N0 patients who presented lymph node metastasis during follow up (submitted to lymphadenectomy) had similar survival to patients without lymph node recurrence (p = 0.219).

**Conclusion:**

Local advanced tumors are at risk of lymph node metastasis. Increased risk is associated to previous lesions at tumor site. T4 classification have worse prognosis. Lymph node recurrences in N0 patients, once treated, did not affect survival. For these patients, we propose close follow up and prompt treatment of lymph node metastasis. These results do not support indication for elective lymphadenectomy or sentinel node mapping. Further prospective studies must address this issue.

## Background

Squamous cell carcinoma of the skin (SCCS) is one of the most common cancers around the word [1,2], with an increasing incidence at a rapid rate among Caucasians in Europe, America and Australia [3-6]. It affects typically white skin populations and usually in body areas under sun exposure. In majority of cases the progression is indolent, and is easily cured by simple excision or radiotherapy.

Different from head and neck skin squamous cell carcinomas that present a higher incidence of lymph node addressed by several studies [7-9], trunk and extremities tumors usually do not present metastasis and the biological behavior is less aggressive [10,11]. Although it may be uncommon for a practicing dermatologist to observe a metastasis, locally advanced tumors, more frequent observed in referral hospitals, may present higher local recurrence and tissue invasion, regional lymphatic and even distant metastasis, consisting in a high risk group [11,12]. Most of those cases have a late progress to lymph node metastasis, with advanced local and regional disease. Nevertheless, at this point, disease control is difficult and survival is poor [10-13].

The frequency of lymph node metastasis is debatable and the reported rates vary considerable among investigators, from 0.1 to 28%, with a resulting mortality from 50–75%. Also, the inclusion of head and neck tumors in those series is common [13,14]. Few studies have addressed specifically the SCCS of the trunk and extremities [10,11,15,16]. Some have looked at several prognostic factors, but most of them are heterogeneous or include also initial stage lesions and head and neck tumors [10-16].

The present study investigates a consecutive series of only localized advanced SCCS of the trunk and extremities, in order to define prognostic factors to lymph node metastasis and survival.

## Methods

Patients with locally advanced SCCS (T3 and T4 classification – AJCC) [17]were identified from the Hospital do Cancer de Barretos database – Barretos – Brazil. Patients with tumors from head and neck or genital origin were excluded. Patients with previous cancer diagnosis with exception to cutaneous basal cell carcinoma were also excluded due the difficulty to diagnosis the possible origin of metastasis or death of cancer. A total of 57 consecutive patients admitted and treated from October 1987 to November 2005 were evaluated. Pathologic diagnosis of SCC was confirmed in all cases. All of them were classified according to the 2002 American Joint Committee on Cancer staging system and have T3 (tumor >5 cm) or T4 (invasive of deep extra-dermal structures) classification. Institutional Review Board approval was obtained and all information retrospectively collected from medical records.

Several clinical variables were examined. These include age, gender, ethnicity, preceding chronic skin lesions(burns, scars, varicosae ulcers and others) at the site of the tumor, patient origin (rural or urban), anatomic site and treatment. Patterns of lymph node metastasis, recurrence and survival outcomes were also recorded.

We classified the patients related to lymph node metastasis as: N0 – patients with no evidence of lymph node metastasis at presentation; N0f – patients with no evidence of lymph node metastasis at any time; N1 – patients with lymph node metastasis at presentation; N1f – patients with lymph node metastasis during follow up.

We considered lymph node metastasis at presentation (N1) or during follow up (N1f) as endpoint. To analyze the association between clinical variables and lymph node metastasis the chi square, Fisher exact and T-test was used. Overall survival (OS) was also studied, and curves were constructed using the Kaplan-Meier method and compared using the log-rank test. All tests were two-sided, and a p-value of ≤ 0.05 was considered statistically significant.

## Results

We included 57 patients in this study. They were 34 men and 23 women; mean age was 61.9 years (range 30–91 years). 48 were Caucasians and 48 came from urban areas. Preceding chronic skin lesions at the site of SCC occurred in 15 cases. The tumors were sited at: the trunk, 14 patients; upper extremity (shoulder, arm and forearm), 7; lower extremity (thigh and leg) 16; hand 12 and foot 8. The T classification was T3 in 36 cases and T4 in 21 cases. 11 patients initially presented with lymph node metastasis (N1).

Surgery was performed in 46 patients; exclusive radiation therapy in 11. Eight patients received adjuvant radiation therapy. Four of them on the area of lymph node metastasis after lymphadenectomy, at discretion of the attending physician. Major concerns were coalescent tumor and more than 4 lymph node metastasis. Other 4 patients received neoadjuvant radiation on the site of primary tumor, with intention of local response and posterior limb sparing surgery, what was possible in one patient. Lymph node dissection was performed in 21 patients, 11 at the time of primary surgery and 10 during the follow up period. No elective lymph node dissection or sentinel node dissection was performed (Table 1). From 46 patients submitted to surgery, 38 had local control (82.6%) and 8 (17.4%) local recurrence. From 11 patients who received radiation therapy, 9 achieved local control. Recurrence occurred in 2 (18.2%). There was no difference between the treatment modalities (p = 1.0).

**Table 1:** Treatment of 57 patients and characteristics of lymph node metastasis

**Treatment**	**Number**	**%**
**Surgery**		
Wide local excision	1	1.8
Graft or flap	21	38.6
Amputation/desarticulation	24	42.1
		
**Radiation therapy**	11	19.3
		
**Characteristics of lymph node metastasis**		
Anatomic site		
Right axilla	4	7.0
Left axilla	7	12.3
Right inguinal groin	4	7.0
Left inguinal groin	7	12.3
None	35	61.4
		
**Time of presentation**		
Initial (N1)	11	19.6
Recurrence (N1f)	11	19.6
None	35	60.8

From all patients, 22 presented nodal disease. 11 of them presented lymph node metastasis at diagnosis (N1), another 11 cases presented lymph node disease during follow up (N1f) and the median time for it be diagnosed was 11.5 months (ranging from 1.6 to 33.8 months). The distribution of the lymph node metastasis in all cases is presented in Table 1. 21 of those patients underwent lymph node dissection, and 6 of them recurred at the lymph node basin.

Of 11 patients who presented lymph node metastasis during follow up, 7 (63.6%) were T3 and 4 (36.4%) were T4. Nine were submitted to lymphadenectomy, 01 was treated with radiotherapy and 01 refused treatment. The treatment of choice of the primary tumor was radiotherapy in 2 (18.2%). Five (45.5%) had surgery with local reconstruction and 4 (36.4%) amputation. All patients achieved local control of the primary tumor.

Considering the other recurrences, local ones occurred in 6 cases and 4 patients presented distant metastasis: one patient had lung metastasis only, 2 had lung and bone metastasis and the other one had disseminated metastatic disease (lung, bone, liver and skin). The untreated patient with lymph node metastasis had advanced local disease and refused any treatment.

At the time of last follow up, 19 patients were alive without evidence of disease, 17 died due to disease and 12 died from other causes. Nine patients were lost of follow up. Actuarial 5-years overall survival was 40.6%.

The only statistically significant clinical variable associated to increased number of lymph node metastasis was the presence of previous lesions on the skin. 9 of 22 patients with lymph node metastasis over the time presented previous lesions, versus 6 who did not (p = 0.047). Lesions located in upper extremity presented more lymph node metastasis, but did not reach statistical significance. 5 of 7 cases presented metastasis versus 17 of 50 from other locations (p = 0.095). Regarding the N0 patients, 11 presented lymph node metastasis during the follow up period (N1f) and again previous lesion was the only statistically significant factor: 5 of 11 patients with preceding chronic skin lesion at the site of SCC presented lymph node metastasis, versus 6 of 35 (p = 0.050). The mean time of presentation of lymph node metastasis in the 11 N1f group was 12.7 months (median, 11.5 months). Yet, the mean follow up of patients that never presented lymph node metastasis over the time (N0f) was 30.6 months (median, 22.7 months) (Table 2).

**Table 2:** Univariate analysis of risk factors for lymph node metastasis

**Variable**	**LN - (N0f) n (%)**	**LN + (N1+N1f) n (%)**	**p value**
**Age (mean)**	64.22	58.38	0.154*
			
**Gender**			0.407^¥^
Male	19 (33.3)	15 (26.3)	
Female	16 (28.1)	7 (12.3)	
			
**Ethnicity**			0.514^†^
Caucasian	31 (54.4)	17 (29.8)	
African	1 (1.7)	1 (1.7)	
			
**Residence**			0.408^¥^
Urban	30 (52.6)	18 (31.6)	
Rural	5 (8.8)	1 (1.7)	
			
**Lesion location**			0.095^¥^
Upper extremity (without hand)	2 (3.5)	5 (8.8)	
Trunk, hand and lower extremity	33 (57.9)	17 (29.9)	
			
**Previous lesion**			0.047^†^
Present	6 (10.5)	09 (15.8)	
Absent	29 (50.9)	13 (22.8)	
			
**Treatment modality**			0.309^¥^
Surgery	27 (47.4)	19 (33.3)	
Radiation therapy	8 (14.0)	3 (5.3)	
			
**T stage**			0.158^¥^
T3	25 (43.8)	11 (19.3)	
T4	10 (17.5)	11 (19.3)	

* T – test

^† ^Chi square

^¥ ^Fisher exact

**Figure 1:**
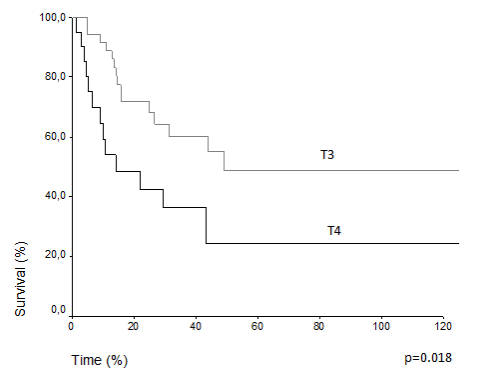
Overall survival for T3 and T4 patients.

**Figure 2:**
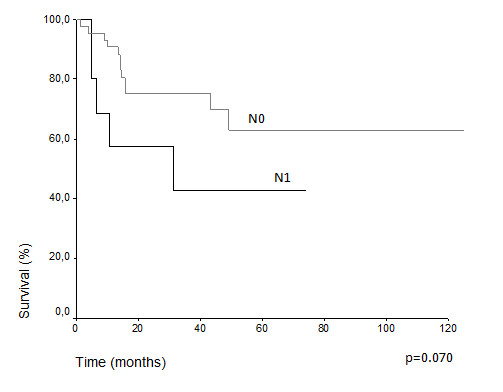
Overall survival for N0 and N1 patients.

**Figure 3:**
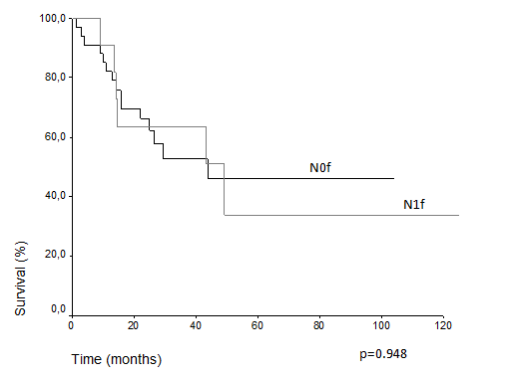
Overall survival for N0 patients considering presence of lymph node metastasis during follow-up.

**Figure 4:**
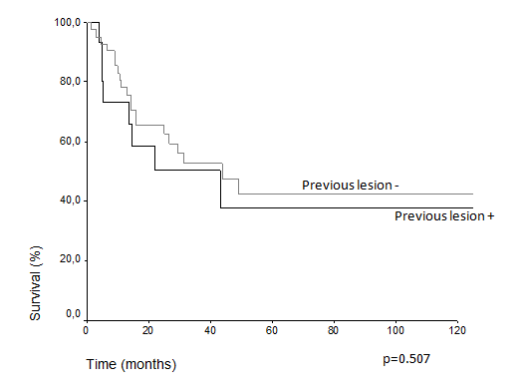
Overall survival considering the presence of previous skin lesions.

Comparative survival curves demonstrate differences in actuarial overall survival among patients with T3 and T4 classification (5 years overall survival 48.7% vs. 24.2%, p = 0.018) (Figure 1). Among N0 and N1, there was difference, but it was not statistically significant (43.3% vs. 34.1%, p = 0.070) (Figure 2). The treatment of choice of the primary tumor did not show differences in overall survival. Actuarial 5-years survival was 42.15% in the surgery group versus 40.91% (p = 0,659) in the radiation group. Overall survival estimative for N0 patients did not show difference regarding the presentation of lymph node metastasis over the time (N1f vs. N0f): (33.9% vs. 46.2%, p = 0.948) (Figure 3) (Table 3). Despite the risk for development of lymph node metastasis, comparative survival did not show difference between patients with previous skin lesions or not (37.7% vs. 42.2%, p = 0.507) (Figure 4).

**Table 3:** Overall survival of 57 patients with advanced SCCS according to clinical variables

**Groups**	**N**	**5-years survival (%)**	**p value**
**T stage**			0.018
**T3**	36	48.7	
**T4**	21	24.2	
**N stage**			0.070
**N0**	46	43.3	
**N1**	11	34.1	
**Anatomic site**			0.378
**Upper extremity**	07	47.6	
**Other**	50	40.0	
**Previous lesion**			0.507
**Present**	15	37.7	
**Absent**	42	42.2	
**Lymph node metastasis**			0.948
**Present (N1f)**	11	33.9	
**Absent (N0f)**	35	46.2	

## Discussion

In our series of 57 locally advanced SCCS of the trunk and extremities, we observed a high frequency of lymph node metastasis. However, univariate analysis identified only one significant risk factor for development of lymph node metastasis, the presence of scar or ulcers previously in the area of the tumor. This was observed also in other studies [10,18]. The association of UV damage and a burn or chronic wound may be associated to the aggressiveness and metastasis in these patients. The anatomic site may be a risk factor (upper extremity presented with more lymph node metastasis), but the number of our cases was not able to show statistical significance. Other studies reported anatomic sites as high risk for metastasis, but with conflicting results, and including head and neck tumors [7,19,20].

The 24 cases of amputations (42.1%) also reflected the selection of local advanced disease. Sixteen of 24 cases (66.7%) were T4. Most of them had no possibility to try other local treatment. Clinical difficulty to major reconstruction was a concern in some cases, like diminished vascular irrigation, old age, cardiovascular diseases or other morbidities.

From our patients, 22 presented lymph node metastasis. The high incidence of metastasis in this selected group, confirms that tumors greater than 5 cm or invasive to soft tissue or bones (T3 and T4) are high risk for lymphatic spread. The 11 patients with metastasis at presentation, also reflects possible referral bias to tertiary cancer center and is similar to other reports [10,15]. Comparative survival demonstrates that more advanced tumors (T4) had worse prognosis with poor outcome. This was expected and confirms the value of T stage from the TNM classification as a prognostic tool. The diagnosis of lymph node metastasis during follow-up in N0 patients occurred in 24.4% of cases. This information would be enough for indication of elective lymphadenectomy or sentinel node dissection in most tumors. However, survival analysis among patients without initial lymph node metastasis (N0), showed no difference considering those who developed lymph node metastasis during the follow up or not (N0f versus N1f). One comment is that all the patients were closely followed, and lymph node disease was treated by lymph node dissection soon after diagnosis. So, similar survival might be explained by the prompt therapy. It is important to advert this was a retrospective study with a relatively small sample with heterogeneous treatment options, submitted to Univariate analyze. The results show no indication for elective lymph node dissection, or sentinel node mapping in these specific setting of patients, since careful surveillance and early surgery after clinical/radiological diagnosis is performed, but this affirmation needs confirmation in prospectively randomized studies. Distant metastasis is not usual even in locally advanced or after lymph node metastasis in SCCS of the trunk and extremities. Only 4 patients had metastasis in distant organs. Despite of this, approximately 1/3 of patients died of disease, mainly due to loco-regional failure, which is the major problem in control disease of these patients.

## Conclusion

Local advanced tumors are at risk of lymph node metastasis. Increased risk is associated to previous lesions at tumor site, and T4 classification tumors have worse prognosis. Lymph node recurrences in N0 patients, once treated, did not affect survival. For these patients, we propose close follow up and prompt treatment of lymph node metastasis. These results do not support indication for elective lymphadenectomy or sentinel node mapping. Further prospective studies must address this issue.

## Competing interests

The authors declare that they have no competing interests.

## Authors' contributions

VLV: Conception, design, analysis of data; TS: Acquisition and analysis of data; NMP: Acquisition and analysis of data; ALC: Interpretation of data, critical review, design. All authors read and approved the final manuscript.
